# Supplementary material for: Osteocytes Influence on Bone Matrix Integrity Affects Biomechanical Competence at Bone-Implant Interface of Bioactive-Coated Titanium Implants in Rat Tibiae
Source: Int J Mol Sci. 2021 Dec 29;23(1):374. doi: 10.3390/ijms23010374 (PMC8745552; doi:10.3390/ijms23010374)
Supplement: Supplementary file 1 [file ijms-23-00374-s001.zip › ijms-1517853-supplementary.pdf]

International Journal of
Molecular Sciences

MDPI

Article

Osteocytes Influence on Bone Matrix Integrity Affects Biomechanical Competence at Bone-Implant Interface of Bioactive-Coated Titanium Implants in Rat Tibiae

Sabine Stoetzel [1,†], Deeksha Malhan [1,†], Ute Wild [1], Christian Helbing [2], Fathi Hassan [1], Sameh Attia [3], Klaus D. Jandt [2], Christian Heiss [1,4] and Thaqif El Khassawna [1,*]

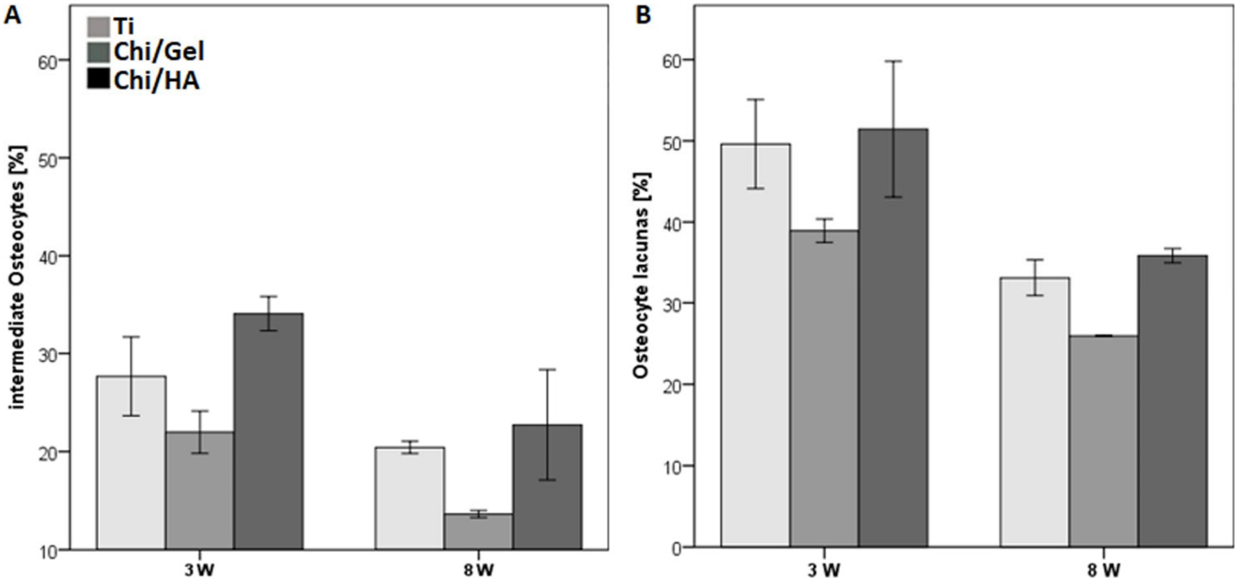

Figure S1. Quantitative evaluation of osteocytes within bone-implant interface showed the less dynamic change in represented as intermediate (round shaped) osteocytes and empty lacunae in all groups after 8W. However, the lowest change was seen in the Chi/Gel group. A) Lower number of intermediate osteocytes were seen in all three groups after 8W compared with 3W. However, higher numbers were seen in the Chi/HA group at both time points compared to the other two groups. B) Lower number of empty lacunae seen in all groups after 8W compared with 3W. However, highest numbers were seen in the Chi/Gel at both time points. (Ti: n = 5 (3W), n = 3 (8W); Chi/Gel: n =5/time point; Chi/HA: n = 4 (3W), n = 5 (8W))

Table S1. Mean ± SEM measurements of osteoblasts and osteoclasts count from modified Masson-Goldner stain.

Cell type	TP	**Ti**<br>Mean ± SEM (μm^2)	**Gel**<br>Mean ± SEM (μm^2)	**HA**<br>Mean ± SEM (μm^2)
**Osteoblasts**	**3W**	2 ± 1	1.3 ± 1.5	3 ± 1.4
	**8W**	5 ± 7	2 ± 2.8	2 ± 1.4
**Osteoclasts**	**3W**	1.7 ± 1.5	0.7 ± 0.6	3 ± 1.4
	**8W**	2.7 ± 3.8	3 ± 0	2 ± 1.4

Table S2. Mean ± SEM measurements of osteocytes count/bone area from Silver Nitrate stain.

**Cell type**	TP	Ti Mean ± SEM (μm^2)	Gel Mean ± SEM (μm^2)	HA Mean ± SEM (μm^2)
**Total osteocytes**	3W	2.87*10-7 ± 1.68*10-7	6.8*10-7 ± 1.78*10-7	3.52*10-7 ± 3.19*10-7
	8W	5.86*10-7 ± 2.73*10-7	9.0*10-7 ± 2.7*10-8	7.64*10-7 ± 8.443*10-8

Table S3. Percentage distribution of spindle shaped osteocytes, intermediate osteocytes, and empty lacunae in bone-implant interface.

Cell type	TP	**Ti** (%)	**Gel** (%)	**HA** (%)
**Spindle shaped osteocytes**	**3W**	22.5 %	39.1 %	14.5 %
	**8W**	46.5 %	60.4 %	42.4 %
**Intermediate osteocytes**	**3W**	27.7 %	22 %	34.1 %
	**8W**	20.4 %	13.6 %	22.7 %
**Empty lacunae**	**3W**	49.2 %	38.9 %	51.4 %
	**8W**	33.1 %	26 %	35.9 %

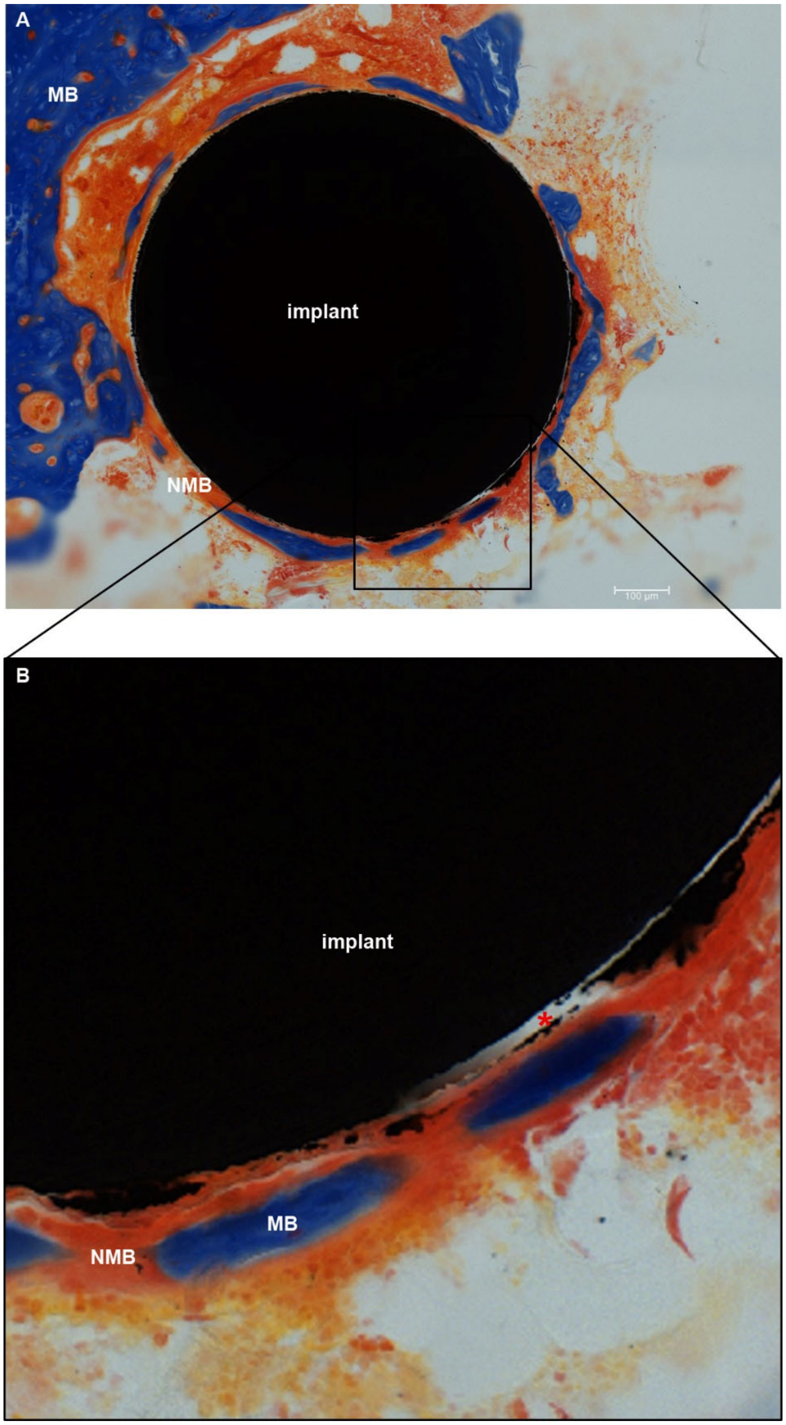

Figure S2: Details of the areas at the bone implant interface. (A) an overview. (B) a close-up to show the main areas. Non-mineralized bone matrix (NMB). Mineralized Bone Matrix (MB) and the asterisks in red indicates an example of void area around the implant.
